# Venous thromboembolism incidence shortens survival in isocitrate dehydrogenase wild-type glioblastoma

**DOI:** 10.1093/noajnl/vdaf018

**Published:** 2025-01-31

**Authors:** Anthony R Sloan, Alan J Gordillo, Austin Kennemer, Alok A Khorana, Craig Horbinski, David C Kaelber, Scott J Cameron, Justin D Lathia

**Affiliations:** Case Comprehensive Cancer Center, Cleveland, Ohio, USA; Lerner Research Institute, Cleveland Clinic, Cleveland, Ohio, USA; Cleveland Clinic Lerner College of Medicine of Case Western Reserve University, Cleveland, Ohio, USA; Lerner Research Institute, Cleveland Clinic, Cleveland, Ohio, USA; Case Western Reserve University School of Medicine, Cleveland, Ohio, USA; Taussig Cancer Institute, Cleveland Clinic, Cleveland, Ohio, USA; Cleveland Clinic Lerner College of Medicine of Case Western Reserve University, Cleveland, Ohio, USA; Case Comprehensive Cancer Center, Cleveland, Ohio, USA; Department of Pathology, Northwestern University, Chicago, Illinois, USA; The Center for Clinical Informatics Research and Education, The MetroHealth System and the Departments of Internal Medicine, Pediatrics, and Population and Quantitative Health Sciences, Case Western Reserve University, Cleveland, Ohio, USA; Department of Cardiovascular Medicine Section of Vascular Medicine, Cleveland Clinic, Cleveland, Ohio, USA; Case Comprehensive Cancer Center, Cleveland, Ohio, USA; Lerner Research Institute, Cleveland Clinic, Cleveland, Ohio, USA; Rose Ella Burkhardt Brain Tumor & Neuro-Oncology Center, Cleveland Clinic, Cleveland, Ohio, USA; Taussig Cancer Institute, Cleveland Clinic, Cleveland, Ohio, USA; Case Comprehensive Cancer Center, Cleveland, Ohio, USA; Lerner Research Institute, Cleveland Clinic, Cleveland, Ohio, USA

**Keywords:** glioblastoma, thrombosis

**Cancer-related thrombosis, specifically venous thromboembolism (VTE) consisting of either one of—or both—deep vein thrombosis or pulmonary embolism (PE), is a common comorbid condition in patients with cancer.^1^ Isocitrate dehydrogenase (IDH) wild-type glioblastoma (GBM) is an underappreciated disease with increased VTE risk. GBM is highly vascularized and produces angiogenic factors, with 92% of GBM resections showing histological evidence of intravascular thrombosis; this is greater than any other central nervous system tumor.^2^ Moreover, thrombosis-related signaling due to aberrant platelet activation has been reported in GBM,^3,4^ underscoring these mechanisms as potential drivers of GBM growth.**

While previous analyses have demonstrated that VTE rates are greater in GBM patients using smaller patient cohorts in high-grade glioma,^5–8^ since the release of the updated fifth edition of the World Health Organization classification, a systematic analysis in a large-scale cohort of patients with IDH-wildtype GBM with clinical outcomes is lacking. To address this unanswered question, we leveraged the Research USA Minimal Date Shift network in the TriNetX platform composed of aggregated, de-identified electronic health record data of 93 million from various healthcare organizations nationwide. Inclusion criteria for general GBM comparisons included the diagnosis of IDH-wildtype GBM. Healthy patient comparison inclusion criteria were an in-patient hospital stay for nonacute causes with no neoplasm diagnosis. Index events were queried such that their occurrence must fall within the years 2000-2023 for 1-year follow-up data and 2000-2019 for 5-year follow-up data. Queries were assessed on October 2024. VTE was selected as any instance of the ICD-10-CM codes I82 (other venous embolism and thrombosis) and I26 (PE). Log-rank analysis was performed using sex-specific Kaplan-Meier survival curve models adjusted for age at index event, sex, and age at diagnosis.

The study population comprised 1535 healthy patients,1496 patients diagnosed with IDH-wildtype GBM with a VTE, and a cohort of 1138 patients with IDH-wildtype GBM with no VTE. Propensity-matched clinical cases were evaluated based on age at index, race, sex, ethnicity, metabolic disorders, hypertension, ischemic heart disease, and diabetes. After propensity-matching, each cohort was well-balanced with standardized differences with *P* < .01. Patients with GBM had a 24% incidence of VTE compared to a 2.3% incidence for in-patient hospitalizations for all causes (OR = 13.3, 95% CI = 9.3-19.0). At 1-year follow-up, GBM patients had a 19.1% rate of VTE compared to 1.394% in the healthy nonneoplastic population (OR = 25.3, 95% CI = 15.0-42.8). At 5 years follow-up, GBM patients had a 21.9% rate of VTE compared to 3.2% in the healthy nonneoplastic population (OR = 7.5, 95% CI = 4.7-12.0).

**Figure 1:**
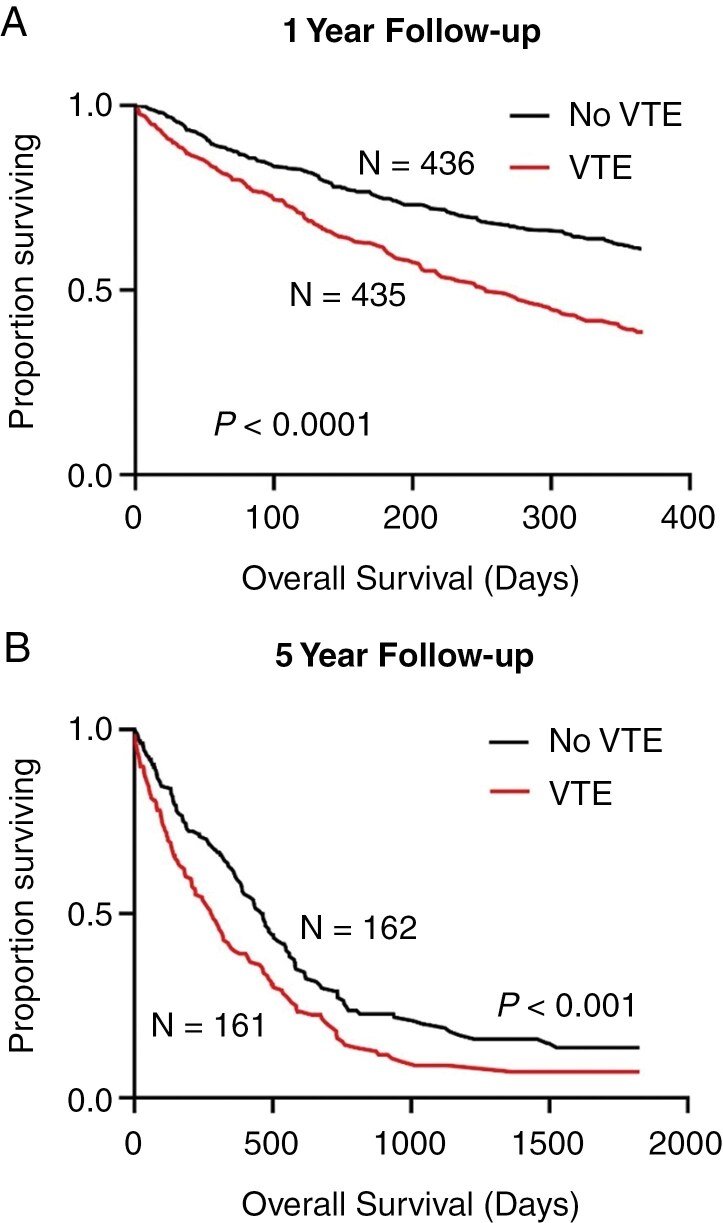
(A) One-year survival of IDH-wt GBM patients diagnosed with a VTE and those with no VTE. (B) Five-year survival of IDH-WT GBM patients diagnosed with a VTE and those with no VTE. GBM, glioblastoma; IDH, isocitrate dehydrogenase; VTE, venous thromboembolism; WT, wild type.

At both the 1 and 5 years following GBM diagnosis, after propensity score matching as described above, patients with a VTE had a significantly higher risk for all-cause mortality compared to patients without a clinical diagnosis of VTE (Figure 1, hazards ratio (HR) = 0.5, 95% CI = 0.4-0.7; HR = 0.6, 95% CI = 0.5-0.94). Of note, there was no significant difference in mortality risk for males and females 1 year following GBM diagnosis or 5 years following GBM diagnosis.

The results of this analysis confirm that more than 20% of IDH-WT GBM patients develop VTE. Separate analysis with unique cohorts was done to compare methylation of the O^6^-methylguanine-DNA methyltransferase (MGMT) promoter status (Methylated: *n = *98, Unmethylated: *n* = 139), sex, and IDH status (IDH mutant: *n* = 1624, IDH wild type *n* = 67), which were not predictive of VTE incidence. We acknowledge that a prior study using the Least absolute shrinkage and selection operator (LASSO) method did indicate a protective effect of IDH and MGMT methylation status.^5^ A limitation of this study—or any study relying on administrative databases—is the possibility of incorrect coding for diagnoses at the time of clinical care.

A limitation to this analysis using the TriNetX platform is the lack of information regarding the cause of mortality, which future studies should address. However, a previous report has demonstrated that although VTE incidence is a leading cause of death in patients with GBM, only 1% of GBM-related deaths are a consequence of VTE, whereas 90% of patients with GBM and a VTE die because of tumor progression.^6^ Mechanistic data are needed to clearly understand how platelets and the coagulation cascade are primed toward a thrombotic state to identify how these mechanisms uniquely contribute to GBM disease progression, identifying therapeutic targets.
